# Integrated genomic study of quadruple-WT GIST (*KIT/PDGFRA/SDH/RAS* pathway wild-type GIST)

**DOI:** 10.1186/1471-2407-14-685

**Published:** 2014-09-20

**Authors:** Margherita Nannini, Annalisa Astolfi, Milena Urbini, Valentina Indio, Donatella Santini, Michael C Heinrich, Christopher L Corless, Claudio Ceccarelli, Maristella Saponara, Anna Mandrioli, Cristian Lolli, Giorgio Ercolani, Giovanni Brandi, Guido Biasco, Maria A Pantaleo

**Affiliations:** Department of Specialized, Experimental and Diagnostic Medicine, Sant’Orsola-Malpighi Hospital, University of Bologna, Via Massarenti 9, 40138 Bologna, Italy; “Giorgio Prodi” Cancer Research Center, University of Bologna, Bologna, Italy; Pathology Unit, S. Orsola-Malpighi Hospital, University of Bologna, Bologna, Italy; Portland VA Medical Center and Knight Cancer Institute, and Division of Hematology and Oncology, Oregon Health & Science University Portland, Portland, OR USA; Department of Pathology and Knight Cancer Institute, Oregon Health & Science University, Portland, OR USA; Transplant, General and Emergency Surgery Department, S.Orsola-Malpighi Hospital, University of Bologna, Bologna, Italy

**Keywords:** Gastrointestinal stromal tumors (GIST), Wild-type, *KIT*, *PDGFRA*, Succinate dehydrogenase, *SDHA*, *RAS*, *Quadruple*^WT^

## Abstract

**Background:**

About 10-15% of adult gastrointestinal stromal tumors (GIST) and the vast majority of pediatric GIST do not harbour *KIT* or platelet-derived growth factor receptor alpha (*PDGFRA*) mutations (J Clin Oncol 22:3813–3825, 2004; Hematol Oncol Clin North Am 23:15–34, 2009). The molecular biology of these GIST, originally defined as *KIT/PDGFRA* wild-type (WT), is complex due to the existence of different subgroups with distinct molecular hallmarks, including defects in the succinate dehydrogenase (*SDH*) complex and mutations of neurofibromatosis type 1 (*NF1*), *BRAF*, or *KRAS* genes (*RAS*-pathway or *RAS-P*).

In this extremely heterogeneous landscape, the clinical profile and molecular abnormalities of the small subgroup of WT GIST suitably referred to as *quadruple wild-type* GIST (*quadruple*^WT^ or *KIT*^WT^/*PDGFRA*^WT^/*SDH*^WT^/*RAS-P*^WT^) remains undefined. The aim of this study is to investigate the genomic profile of *KIT*^WT^/*PDGFRA*^WT^/*SDH*^WT^/*RAS-P*^WT^ GIST, by using a massively parallel sequencing and microarray approach, and compare it with the genomic profile of other GIST subtypes.

**Methods:**

We performed a whole genome analysis using a massively parallel sequencing approach on a total of 16 GIST cases (2 *KIT*^WT^/*PDGFRA*^WT^/*SDH*^WT^ and *SDHB*^IHC+^/*SDHA*^IHC+^, 2 *KIT*^WT^/*PDGFRA*^WT^/*SDHA*^mut^ and *SDHB*^IHC-^/*SDHA*^IHC-^ and 12 cases of *KIT*^mut^ or *PDGFRA*^mut^ GIST). To confirm and extend the results, whole-genome gene expression analysis by microarray was performed on 9 out 16 patients analyzed by RNAseq and an additional 20 GIST patients (1 *KIT*^WT^/*PDGFRA*^WT^*SDHA*^mut^ GIST and 19 *KIT*^mut^ or *PDGFRA*^mut^ GIST). The most impressive data were validated by quantitave PCR and Western Blot analysis.

**Results:**

We found that both cases of *quadruple*^WT^ GIST had a genomic profile profoundly different from both either *KIT/PDGFRA* mutated or *SDHA*-mutated GIST. In particular, the *quadruple*^WT^ GIST tumors are characterized by the overexpression of molecular markers (*CALCRL* and *COL22A1*) and of specific oncogenes including tyrosine and cyclin- dependent kinases (*NTRK2* and *CDK6*) and one member of the *ETS*-transcription factor family (*ERG*).

**Conclusion:**

We report for the first time an integrated genomic picture of *KIT*^WT^/*PDGFRA*^WT^/*SDH*^WT^/*RAS-P*^WT^ GIST, using massively parallel sequencing and gene expression analyses, and found that *quadruple*^WT^ GIST have an expression signature that is distinct from *SDH*-mutant GIST as well as GIST harbouring mutations in *KIT* or *PDGFRA*. Our findings suggest that *quadruple*^WT^ GIST represent another unique group within the family of gastrointestintal stromal tumors.

**Electronic supplementary material:**

The online version of this article (doi:10.1186/1471-2407-14-685) contains supplementary material, which is available to authorized users.

## Background

**Figure 1:**
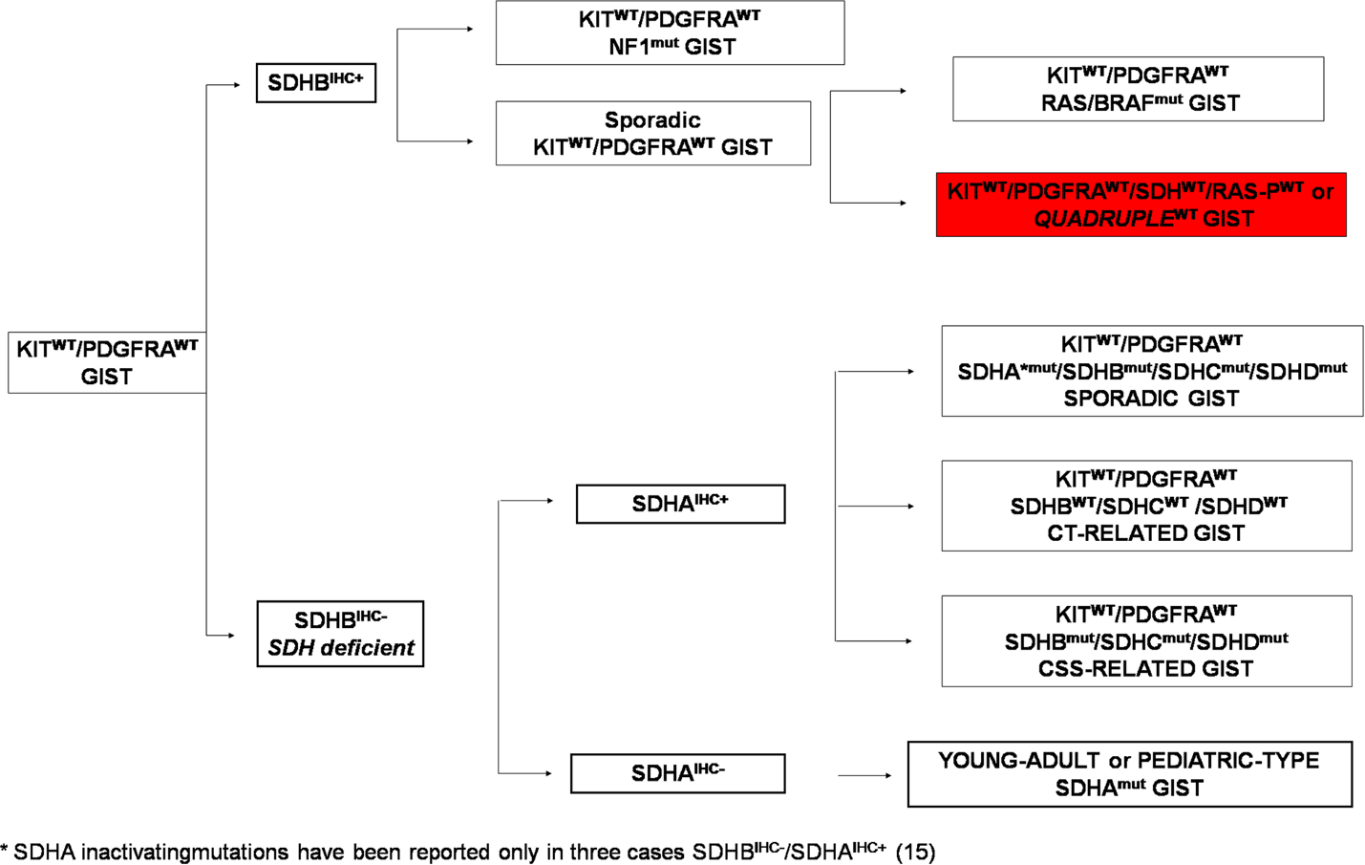
**The complexity of** ***KIT*** ^**WT**^ **/** ***PDGFRA*** ^**WT**^ **GIST molecular biology.** *KIT* ^WT^/*PDGFRA* ^WT^ GIST could be firstly divided two main group according to the *SDHB* immunohistochemical status: *SDHB* ^IHC+^ (including *NF1*-mutated GIST and sporadic *KIT* ^WT^/*PDGFRA* ^WT^ GIST with or without *KRAS/BRAF* mutations) and *SDHB* ^IHC-^ or *SDH-deficient* GIST. The latter could be further divided according to the SDHA immunohistochemical status: *SDHB* ^IHC-^/*SDHA* ^IHC-^ GIST (pediatric type or young adult GIST characterized by germline or somatic inactivating *SDHA* mutations) and *SDHB* ^IHC^/*SDHA* ^IHC+^ GIST (including Carney-Stratakis Syndrome-related GIST, characterized by germline or somatic inactivating *SDHB*, −*C*, −*D* mutations, Carney Triad-related GIST that lack *SDHx* mutations, and sporadic *KIT* ^WT^/*PDGFRA* ^WT^ GIST, characterized by germline or somatic inactivating *SDHB*, −*C*, −*D* mutations and *SDHA* mutations, reported in only three cases [15]. In red the subset of *KIT* ^WT^/*PDGFRA* ^WT^ GIST referred to as *quadruple* ^WT^ GIST (*KIT* ^WT^/*PDGFRA* ^WT^/*SDH* ^WT^/*RAS-P* ^WT^), that represent the subject of this study.

About 10-15% of adult gastrointestinal stromal tumors (GIST) and the vast majority of pediatric GIST do not harbour *KIT* or platelet-derived growth factor receptor alpha (*PDGFRA*) mutations [1, 2]. These GIST were originally defined as *KIT/PDGFRA* wild-type (*KIT*^WT^/*PDGFRA*^WT^) and generally are less sensitive to tyrosine-kinase inhibitors [3–5]. Their molecular biology is heterogeneous as evidence by the existence of different subgroups with distinct molecular abnormalities (Figure 1). *KIT*^WT^/*PDGFRA*^WT^ GIST can be divided into two main groups according to the succinate dehydrogenase subunit B (SDHB) immunohistochemical status (IHC): *SDHB* positive (*SDHB*^IHC+^), or *type 1* GIST which, includes neurofibromatosis type 1 (*NF1*)-mutated GIST and some sporadic *KIT*^WT^/*PDGFRA*^WT^ GIST. The second group of *KIT*^WT^/*PDGFRA*^WT^, called as *type 2* GIST, is characterized by a lack of *SDHB* protein expression (*SDHB*^IHC-^). In some cases *SDHB*^IHC-^ is due to germline and/or *de novo* mutations of any of the four *SDH* subunits (*SDHA*^mut^) [6–8]. The *SDHB*^IHC-^ includes additional subgroups that can be distinguished on the basis of the *SDHA* IHC status, which strictly correlates with the presence of *SDHA*-inactivating mutations (*SDHA*^mut^) [9–16]. In particular, *SDHB*^IHC-^/*SDHA*^IHC-^ GIST include a subgroup of young adult women patients with a well defined clinical and biological profile, generally characterized by the gastric primary tumour localization, a predominantly mixed epithelioid and spindle cell morphology, diffuse IHC positivity for *KIT* and discovered on gastrointestinal stromal tumours 1 (*DOG1*), frequent lymph node metastases, and an indolent course of disease even if metastasis is present [17]. Moreover, they are characterized by overexpression of the insulin growth factor 1 receptor (*IGF1R*) [18–21]. On the contrary, *SDHB*^IHC-^, but *SDHA*^IHC+^ subgroup include 1) cases of syndromic GIST arising from the Carney-Stratakis Syndrome (CSS), that are characterized by *SDHB*, *SDHC* or *SDHD* inactivating mutations (*SDHB*^mut^, *SDHC*^mut^, or *SDHD*^mut^); and 2) cases of Carney Triad (CT), that lack *SDH*x-mutations [6, 22–24]. More rarely, *SDHB*^IHC-^/*SDHA*^IHC+^ subgroup may include sporadic *KIT*^WT^/*PDGFRA*^WT^ GIST characterized by *SDHB*, −*C* or *D* mutations (most of them germline, and in few cases by *SDHA* mutations), arising mainly from the stomach, with a lesser female prevalence, but histologically similar to *SDHA*^IHC-^ GIST [15].

The *SDHB*^IHC+^ subgroup includes cases of *NF1*-mutated GIST, that are commonly intestinal, multifocal and have an *IGF1R* negative staining, and also sporadic *KIT*^WT^/*PDGFRA*^WT^ GIST, arising in the adult from any part of gastrointestinal tract [15, 21, 25]. In about 15% of cases of sporadic *KIT*^WT^/*PDGFRA*^WT^ GIST there may be an activating mutation in *BRAF* or, more rarely, *RAS* [26–28]. Taken together, cases of *BRAF*, *RAS*, or *NF1* mutant GIST can be referred to as *RAS*-pathway (*RAS-P*) mutant GIST (*RAS-P*^mut^).

In this extremely heterogeneous landscape, the clinical profile and molecular abnormalities of the small subgroup of WT GIST suitably referred to as *quadruple wild-type* GIST (*quadruple*^WT^ or *KIT*^WT^/*PDGFRA*^WT^/*SDH*^WT^/*RAS-P*^WT^) remains undefined [29]. The aim of this study is to investigate the genomic profile of *KIT*^WT^/*PDGFRA*^WT^/*SDH*^WT^/*RAS-P*^WT^ GIST, by using a massively parallel sequencing and microarray approach, and compare it with the genomic profile of other GIST subtypes.

## Results and discussion

### Whole-Transcriptome Paired-End RNA Sequencing and copy number analysis

**Figure 2:**
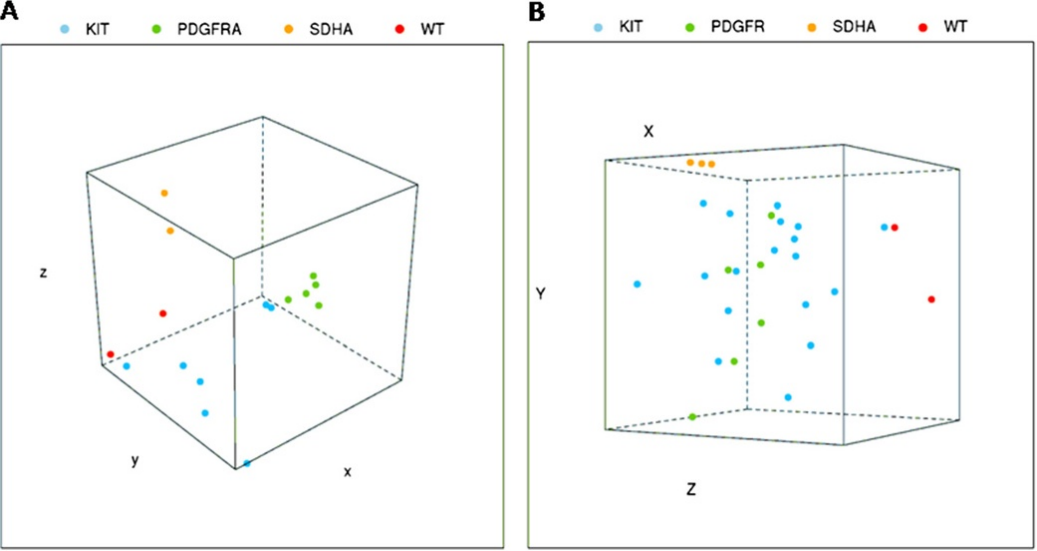
**Principal Component Analysis (PCA) performed on samples analyzed with RNA-seq (Figure** 2 **A) and microarray (Figure** 2 **B).** In both cases the patients with *SDHA* mutations are arranged in a strongly separated cluster (yellow points), as were the *KIT* ^WT^/*PDGFRA* ^WT^/*SDH* ^WT^/*RAS-P* ^WT^ samples (red point) although closer to *KIT* or *PDGFRA* mutated (respectively blue and green point).

Whole-Transcriptome Paired-End RNA Sequencing was performed on a total of 16 GIST samples, of which 2 were *KIT*^WT^/*PDGFRA*^WT^ without *SDH*-inactivating mutations and *SDHB*^IHC+^/*SDHA*^IHC+^ (GIST_133 and GIST_127), 2 were *KIT*^WT^/*PDGFRA*^WT^/*SDHA*^mut^ and *SDHB*^IHC-^/*SDHA*^IHC-^ (GIST_7 and GIST_10), and 12 were *KIT*^mut^ or *PDGFRA*^mut^. The principal component analysis showed that both GIST_133 and GIST_127 (*KIT*^WT^/*PDGFRA*^WT^/*SDH*^WT^ and *SDHB*^IHC+^/*SDHA*^IHC+^) are characterized by a gene expression profile profoundly different from both GIST_7 and GIST_10 (*KIT*^WT^/*PDGFRA*^WT^/*SDHA*^mut^ and *SDHB*^IHC^/*SDHA*^IHC^), while clustering in proximity of a subset of *KIT*^mut^ or *PDGFRA*^mut^ GIST (Figure 2A).

To investigate the presence of novel mutations or small ins/del in the whole coding regions of *KIT* and PDGFRA we analyzed whole transcriptome sequencing data for single nucleotide variant (SNV) and found no private or cryptic mutations. Moreover, no *NF-1*, *BRAF*, *RAS* mutations were found by whole transcriptome sequencing. Therefore, the GIST from these two patients were *KIT*^WT^/*PDGFRA*^WT^/*SDH*^WT^/*RAS-P*^WT^, or *quadruple*^WT^ GIST. Analysis of deleterious mutations from whole transcriptome sequencing did not identify any known oncogenic event or shared alteration in the two patients (Additional file 1: Table S1). Copy number analysis was performed on the two *KIT*^WT^/*PDGFRA*^WT^/*SDH*^WT^/*RAS-P*^WT^ GIST: GIST_133 showed no genomic imbalances, while GIST_127 harbors several macroscopic cytogenetic alterations, including loss of chromosome arms 14q and 22q frequently observed in *KIT/PDGFRA* mutated GIST.

### Gene expression analysis

**Figure 3:**
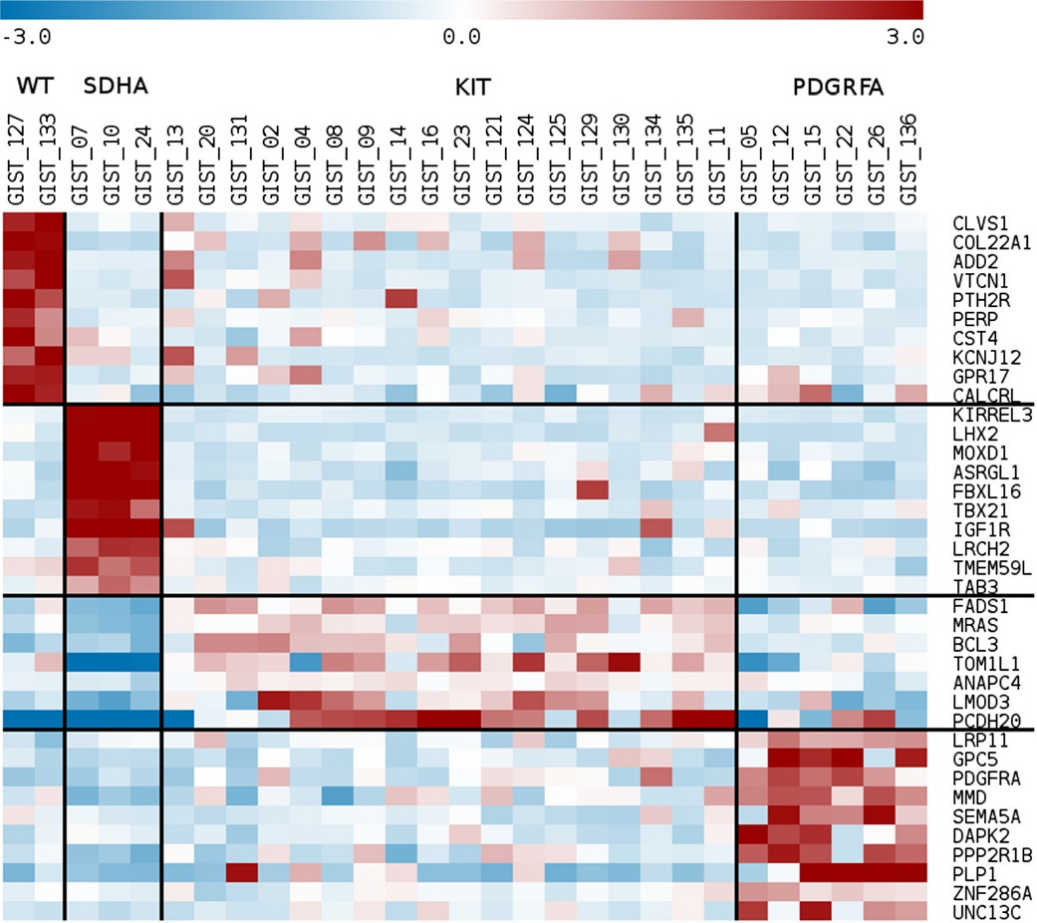
**Representation of top-scoring genes significantly over-expressed in the four GIST classes, (** ***KIT*** ^**WT**^ **/** ***PDGFRA*** ^**WT**^ **/** ***SDH*** ^**WT**^ **/** ***RAS-P*** ^**WT**^ **,** ***SDHA*** ^**mut**^ **,** ***KIT*** ^**mut**^ **and** ***PDGFRA*** ^**mut**^ **), when each of them is compared with all other cases together.**

To confirm and extend the results, whole-genome gene expression analysis by microarray was performed on 9 out 16 patients analyzed by RNAseq and an additional 20 GIST patients (1 *KIT*^WT^/*PDGFRA*^WT^*SDHA*^mut^ GIST and 19 *KIT*^mut^ or *PDGFRA*^mut^ GIST). The principal component analysis confirmed that both *KIT*^WT^/*PDGFRA*^WT^/*SDH*^WT^/*RAS-P*^WT^ GIST have a genetic profile significantly different from all three *KIT*^WT^/*PDGFRA*^WT^/*SDHA*^mut^ GIST, and cluster in close proximity to some *KIT*^mut^ GIST samples (Figure 2B). Supervised gene expression analysis revealed the presence of specific genetic signatures characterizing the different molecular subgroups of GIST (Figure 3); the *SDHA*^mut^ group showed a gene signature mainly characterized by the over-expression of *IGF1R* (p value 2.7X10^−11^) and of neural markers (*LHX2*, *KIRREL3*) [30], whereas as expected, all *PDGFRA*^mut^ GIST were clearly separated from *KIT*^mut^ GIST, especially for the expression of *PDGFRA*.

**Figure 4:**
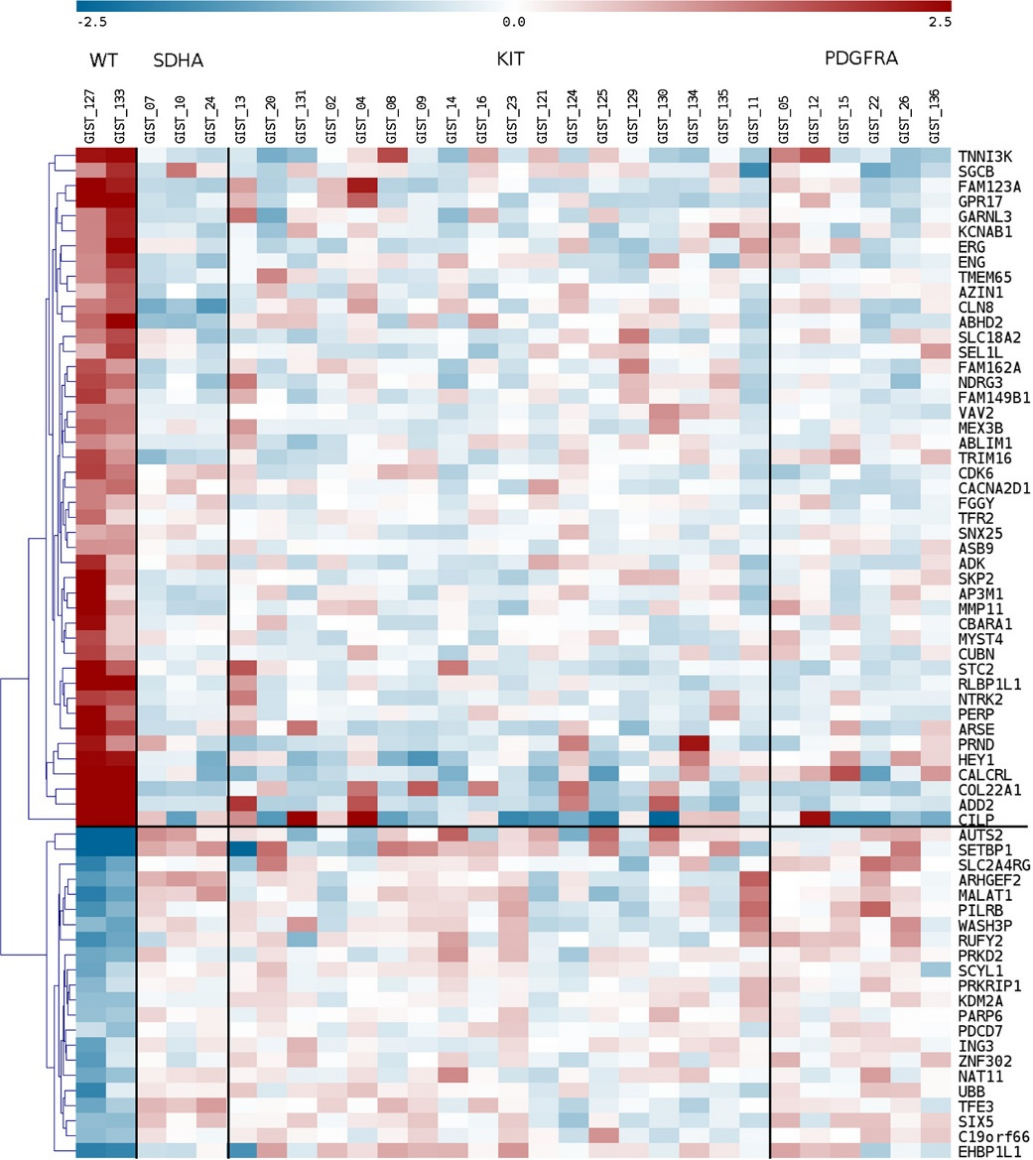
**Unsupervised hierarchical clustering representation of differential expressed genes (P-value < 0.005) in** ***KIT*** ^**WT**^ **/** ***PDGFRA*** ^**WT**^ **/** ***SDH*** ^**WT**^ **/** ***RAS-P*** ^**WT**^ **GIST with respect to the other GIST classes (** ***SDHx*** ^**mut**^ **,** ***KIT*** ^**mut**^ **and** ***PDGFRA*** ^**mut**^ **).**

**Figure 5:**
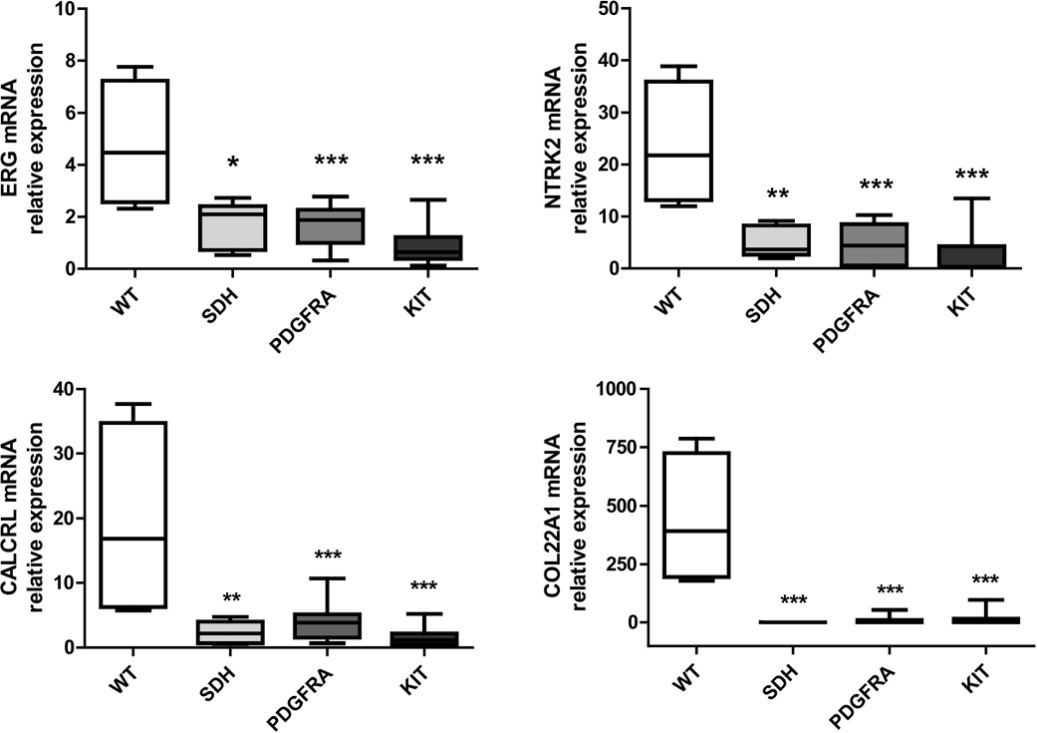
**Quantitative PCR estimation of** ***ERG*** **,** ***NTRK2*** **,** ***CALCRL*** **and** ***COL22A1*** **expression in GIST.** Relative expression of *ERG* (upper left panel), *NTRK2* (upper right panel), *CALCRL* (lower left panel) and *COL22A1* (lower right panel) mRNA in the two *KIT* ^WT^/*PDGFRA* ^WT^/*SDH* ^WT^/*RAS-P* ^WT^ GIST in respect to the others molecular subgroups (4 *SDHx* ^mut^, 19 *KIT* ^mut^ and 10 *PDGFRA* ^mut^ GIST). Significance was estimated by the Student T-test: *p-value < 0.05; **p-value < 0.01; ***p-value < 0.001.

The *quadruple*^WT^ (*KIT*^WT^/*PDGFRA*^WT^/*SDH*^WT^/*RAS-P*^WT^) samples were characterized by a distinct gene expression profile (Figure 4), with 65 genes over-expressed or under-expressed (p value < 0.005) compared with all the other GIST molecular subgroups. GSEA analysis of the transcriptional profile of *quadruple*^WT^ tumors showed enrichment of Polycomb target genes with respect to *SDHA*^mut^ GIST, in particular of the classes of PRC2 targets (p value 0.043) and H3K27-bound genes (p value 0.021). The function of the upregulated genes was related to cell cycle progression and MAPK signaling, ad exemplified by increased expression of SKP2, CDK6, FGF4, NTRK2). The *quadruple*^WT^ GIST tumors are characterized by the overexpression of molecular markers (*CALCRL* and *COL22A1*) and of specific oncogenes including tyrosine and cyclin- dependent kinases (*NTRK2* and *CDK6*) and one member of the *ETS*-transcription factor family (*ERG*). Overexpression of *CALCRL*, *COL22A1*, *NTRK2* (*TrkB*) and of the *ETS*-transcription factor *ERG* was confirmed by quantitative PCR, showing that only the *KIT*^WT^/*PDGFRA*^WT^/*SDH*^WT^/*RAS-P*^WT^ GIST subgroup expressed these molecular markers and possible therapeutic targets (Figure 5). *NTRK2* protein expression level was also evaluated by Western Blot analysis and its overexpression in *quadruple*^WT^ GIST was confirmed (Additional file 2: Figure S1). No mutations, gene fusions or amplifications were identified in *NTRK2* and *ERG*.

## Discussion

The pathogenesis and underlying biology of *KIT*^WT^/*PDGFRA*^WT^ with intact *SDH* complex (*SDHx*^WT^) and non-mutated *RAS*-pathway members (*RAS-P*^WT^) suitably referred to as *quadruple*^WT^ GIST remains undefined. In the present study, we performed a whole genome analysis using a massively parallel sequencing approach on a total of 16 GIST cases that included 2 *KIT*^WT^/*PDGFRA*^WT^/*SDH*^WT^ and *SDHB*^IHC+^/*SDHA*^IHC+,^, 2 *KIT*^WT^/*PDGFRA*^WT^/*SDHA*^mut^ and *SDHB*^IHC-^/*SDHA*^IHC-^ and 12 cases of *KIT*^mut^ or *PDGFRA*^mut^ GIST. Notably, we found that both cases of *quadruple*^WT^ GIST had a transcriptome profile profoundly different from both *KIT/PDGFRA* mutated and *SDHA*-mutated GIST, suggesting a different molecular background underlying *quadruple*^WT^ GIST. Since both cases of *KIT*^WT^/*PDGFRA*^WT^/*SDH*^WT^ lacked mutations of *BRAF*, *RAS* family members or *NF1*, the GIST of these two patients was classified *KIT*^WT^/*PDGFRA*^WT^/*SDH*^WT^/*RAS-P*^WT^ or *quadruple*^WT^ GIST. We further validated our data using genome wide gene expression analysis, performed on 9 cases from a previous series that was expanded to include an additional 20 GIST cases (1 *KIT*^WT^/*PDGFRA*^WT^/*SDHA*^mut^ GIST and 19 *KIT*^mut^ or *PDGFRA*^mut^ GIST). This larger analysis confirmed the unique gene expression signature of the two *quadruple*^WT^ GIST compared to KIT mutant, PDGFRA mutant or SDHA-mutant GIST. Interestingly, the gene signatures of the *quadruple*^WT^ GIST, which both arose in the small intestine, clustered in close proximity to a single *KIT*^mut^ GIST sample (GIST_13). This case was a small intestine GIST of intermediate risk of relapse radically resected from a 46 year old; it harbored an exon 11 *KIT* point mutation (*KIT* exon 11 V559D). Our current sample size does not allow us to draw definitive conclusions, but we hypothesize that the intestinal origin of all three tumors may have influenced the gene signature. However, several other cases of small intestinal origin did not cluster near the cases of *quadruple*^WT^ GIST. The influence of the tissue of origin on the gene signature is consistent with the recent data by Beadling *et al*., who described five cluster groups among 136 GIST patients (53 *KIT*^mut^, 12 *PDGFRA*^mut^, 65 adult *KIT*^WT^/*PDGFRA*^WT^ and 7 pediatric *KIT*^WT^/*PDGFRA*^WT^) defined by the expression patterns of 14 target genes, that were in some cases paralleled by the location of the primary tumour [31].

Using a supervised analysis, we found four gene cluster subgroups based on *KIT/PDGFRA/SDH*-mutational status. Due to the rarity of *RAS-P* mutated GIST, we did not have any cases suitable for these genomic studies. Consistent with previous reports, *KIT*^WT^/*PDGFRA*^WT^/*SDHA*^mut^ GIST over-expressed *IGF1R*, further confirming the potential role of this receptor as a target or diagnostic marker for this specific molecular subgroup [18–21]. Moreover, as already described, the gene signature of *KIT*^WT^/*PDGFRA*^WT^/*SDHA*^mut^ GIST was largely characterized by the expression of neural-commitment transcription markers, in support of the theory that this subgroup may have a different cellular origin or may derive from interstitial cells of Cajal (ICCs) during a different differentiation step, such as from precursors of ICCs [30]. Notably, both *quadruple*^WT^ GIST had a distinct gene expression signature that was separated from the *KIT*^WT^/*PDGFRA*^WT^/*SDHA*^mut^ GIST. Amongst the differentially expressed genes, it is interesting to note the over-expression of *CALCRL*, a G protein-coupled receptor that acts as a receptor for adrenomedullin and calcitonin gene-related peptide (*CGRP*), and is strongly expressed by in several vascular tumours and types of gliomas [32–35]. Also of interest, we found over-expression of *COL22A1*, a member of the collagen protein family which specifically localizes to tissue junctions and acts as a cell adhesion ligand for skin epithelial cells and fibroblasts [36]. Taken together, these findings may suggest the potential role of *CALCRL* and *COL22A1* as diagnostic markers for the identification of this GIST subgroup. This would need to be validated in a larger series of GIST.

We found that both *quadruple*^WT^ GIST, in comparison with the other samples, strongly expressed several oncogenes, including *ERG* and *NTRK2* (*TrkB*). This was confirmed by quantitative PCR. *ERG* is a well-known member of the erythroblast transformation-specific (*ETS*) family of transcription factors, which function as transcriptional regulators [37]. *ETS* proteins are regulated by the mitogenic (*RAS/MAPK*) signalling transduction pathway, and play an important role in cell differentiation, proliferation, apoptosis and tissue remodelling [38]. There is evidence for an oncogenic role of *ERG* and the other *ETS* transcription factors in many human cancers, including sarcomas, prostate cancer, and acute myeloid leukemia, in most cases via chromosomal translocations [39–41]. More recently, it has been shown that the IHC detection of *ERG* may be a useful marker for vascular tumors, prostate carcinoma and *ERG*-rearranged Ewing sarcoma [42–44]. Over-expression of *NTRK2* (*TrkB*) in *quadruple*^WT^ GIST is also of interest, as *NTRK2* helps regulated neuronal cell function, including synaptic plasticity, differentiation, growth, survival, and motility [45]. It has also been shown that *Trks* regulate important processes in non-neuronal cells, contributing to the pathogenesis of several kinds of cancer, such as medullary thyroid carcinoma, prostate cancer, non-small cell lung cancer, head and neck squamous cell carcinoma and pancreatic cancer, in addition to tumors of neural origin [46–51]. Given the relevant biological role played by *Trks* in cancer, different small molecule inhibitors have been developed and evaluated both in mono-therapy and in combination with chemotherapy in phase 1 and 2 clinical trials [52–58].

To our knowledge, the over-expression of *ERG* and *TrkB* in GIST has not been previously reported. However, it is well known that *ETV1*, another member of *ETS* family, is highly expressed in GIST and certain subsets of ICC. *ETV1* expression plays an important role in regulating the growth of *KIT* mutant GIST cell lines [59]. On the basis of our results, the overexpression of *ERG* and *TrkB* seems to be a unique feature of the *quadruple*^WT^ GIST, suggesting that it could play a relevant role in the pathogenesis of this subset of GIST. To translate these observations into clinical practice, the over-expression of both molecules could be investigated as diagnostic markers of *quadruple*^WT^ GIST.

## Conclusions

In conclusion, we report for the first time an integrated genomic picture of the *quadruple*^WT^ GIST, using massively parallel sequencing and gene expression analyses, and have identified a unique subset of GIST among the family of the KIT/PDGFRA WT GIST [60]. The frequency of this GIST subset amongst the family of GIST will need to be defined in future studies as well as any unique clinical-pathological features of this GIST subset, including response to conventional GIST medical therapy. In addition, ongoing studies of ICC developmental biology may help identify the “normal” precursor cells that give rise to this unique GIST subgroup.

## Methods

This study was approved by the institutional review board of Azienda Ospedaliero-Universitaria Policlinico S.Orsola-Malpighi, Bologna, Italy (approval number 113/2008/U/Tess). All patients provided written informed consent.

### Patients and tumor samples

Fresh tissue specimens of GIST from 36 patients were collected during the surgical operation, snap-frozen in liquid nitrogen and stored at −80°C until analysis. Patient’s characteristics are listed in Table 1.

**Table 1:** **Patient’s characteristic**

ID	Sex	Array	RNAseq	Age	Site	Disease status at diagnosis	***KIT/PDGFRA/SDH***mutational status
**GIST_133**	M	X	X	57	Duodenum	Localized	WT
**GIST_127**	F	X	X	63	Ileum	Localized	WT
**GIST_07**	F	X	X	27	Stomach	Metastatic	*SDHA* exon 9 p.S384X
**GIST_10**	M	X	X	29	Stomach	Metastatic	*SDHA* exon 2 p.R31X;
							*SDHA* exon 13 p.R589W
**GIST_188**	F		X	57	Duodenum	Metastatic	*KIT* exon 11 p.N564-L576 del + *KIT* exon 17 p.N822K
**GIST_174**	M		X	59	Stomach	Metastatic	*KIT* exon 11 p.N564_L576 del + *KIT* exon 17 p.N822K
**GIST_131**	M	X	X	58	Ileum	Localized	*KIT* exon 11 p.V569_Y578 del
**GIST_11**	M	X	X	65	Stomach	Localized	*KIT* exon 11 p.557-558 del
**GIST_134**	F	X	X	65	Stomach	Localized	*KIT* exon p.V559D
**GIST_124**	M	X	X	70	Stomach	Localized	*KIT* exon 11 p.1765-1766 ins
**GIST_150**	F		X	55	Stomach	Localized	*KIT* exon 11 p.P551_E554 del
**GIST_165**	M		X	50	Stomach	Localized	*PDGFRA* exon 18 p.D842V
**GIST_136**	M	X	X	76	Stomach	Localized	*PDGFRA* exon 18 p.D842V
**GIST_140**	F		X	45	Stomach	Localized	*PDGFRA* exon 18 p.D842V
**GIST_141**	M		X	68	Stomach	Localized	*PDGFRA* exon 18 p.D842V
**GIST_138**	F		X	75	Stomach	Localized	*PDGFRA* exon 18 p.D842V
**GIST_02**	F	X		85	Stomach	Localized	*KIT* exon 11 p.V560D
**GIST_04**	M	X		79	Stomach	Localized	*KIT* exon 9 p.AY502-503 ins
**GIST_05**	M	X		68	Stomach	Localized	*PDGFRA* exon 12 p.SPDGHE566-571RIQ
**GIST_08**	M	X		62	Stomach	Localized	*KIT* exon 11 p.V559D
**GIST_09**	M	X		54	Stomach	Localized	*KIT* exon 11 TLQPYDHKWEEFP 574–585 ins at P585
**GIST_12**	F	X		66	Stomach	Localized	*PDGFRA* exon 14 p.K646E
**GIST_13**	M	X		46	Small intestine	Localized	*KIT* exon 11 p.V559D
**GIST_14**	M	X		56	Stomach	Localized	*KIT* exon 11 p.WK557-558del
**GIST_15**	F	X		64	Stomach	Localized	*PDGFRA* exon 18 DIMH p.842-845 DIMH del
**GIST_16**	F	X		62	Stomach	Localized	*KIT* exon 11 p.L576P
**GIST_20**	M	X		38	Small intestine	Metastatic	*KIT* exon 11 del MYEQW552-557 Z + *KIT* exon 18 A829P
**GIST_22**	F	X		76	Stomach	NA	*PDGFRA* exon 18 p.D842V
**GIST_23**	F	X		47	Stomach	NA	*KIT* exon 11 p.V559D
**GIST_24**	F	X		18	Stomach	Metastatic	*SDHA* exon 8 p.L349R fs*11
**GIST_26**	M	X		49	Stomach	Localized	*PDGFRA* exon 12 p.V561D
**GIST_121**	M	X		71	Stomach	Localized	*KIT* exon 11 p.V559D
**GIST_125**	F	X		48	Stomach	Localized	*KIT* exon 11 p.W557R
**GIST_129**	M	X		59	Stomach	Localized	*KIT* exon11 p.Y553_V559 delins L
**GIST_130**	F	X		79	Stomach	Localized	*KIT* exon 9 p.A502-Y503 ins
**GIST_135**	F	X		61	Stomach	Localized	*KIT* exon 11 p.W557-E561 del

Whole-Transcriptome Paired-End RNA Sequencing was performed on 16 GIST, including 2 *KIT*^WT^/*PDGFRA*^WT^ GIST patients without *SDH*-inactivating mutations (GIST_133 and GIST_127), 2 *KIT*^WT^/*PDGFRA*^WT^ GIST patients harbouring *SDHA*-mutations (GIST_7 and GIST_10), and 12 *KIT* or *PDGFRA* mutated GIST patients (7 harboured exon 11 *KIT* mutations and 5 harboured exon 18 *PDGFRA* mutations).

Whole-genome gene expression analysis was performed on 9 of the above 16 GIST and extended to include an additional 20 GIST: 1 *KIT*^*W*T^/*PDGFRA*^WT^/*SDHA*^mut^ GIST and 19 *KIT* or *PDGFRA* mutated GIST, of which 13 harboured *KIT* mutations (12 in exon 11 and 2 in exon 9), and 5 harboured *PDGFRA* mutations (2 in exon 12, 1 in exon 14 and 2 in exon 18).

### SDH status

*SDH* protein expression status was evaluated by both immunohistochemistry (IHC) of *SDHB* and *SDH* subunits sequencing. IHC was performed on 4-μm sections of FFPE GIST tumor samples. Rabbit polyclonal anti-*SDHB* (HPA002868, Sigma-Aldrich, St Louis, MO, USA, 1:800) antibody was used. The sections were deparaffinized, rehydrated, and subjected to the appropriate antigen retrieval treatment (*SDHB*: microwave heating in citrate buffer pH 6.0 at 100 1C for 40 min). After cooling at room temperature, the activity of endogenous peroxidises was inhibited using methanol/H2O2 (0.5% v/v) for 20 min. The sections were then washed in phosphate-buffered saline (PBS, pH 7.2–7.4) and incubated with the specific primary antibody overnight at room temperature. After that, the sections were washed in PBS and treated using the Novolink Polymer Detection System (Novocastra, Newcastle upon Tyne, UK) according to the manufacturer’s instructions. Liver tissues (for *SDHB*) were used as positive controls. These tissues showed strong granular staining in the cytoplasm and mitochondria with both of the antibodies.

*SDHA* gene exons [1–15], *SDHB* gene exons [1–8], *SDHC* (exon 1–6) and *SDHD* (exon 1–4) were sequenced on fresh-frozen tumor specimens of *KIT*^WT^/*PDGFRA*^WT^ GIST patients by Sanger Sequencing method. DNA was extracted by the QIAmp DNA Mini kit (Qiagen, Milan, Italy) in accordance with manufacturer’s directions. Each exon was amplified with Polymerase Chain Reaction (PCR) amplification using specific primer pairs designed with Primer Express 3.0 Software (Applied Biosystem) to amplify exons but not *SDHA* pseudo-genes located on chromosomes 3 and 5. Then, PCR products were purified with the Qiaquick PCR purification kit (Qiagen, Milan, Italy) and sequenced on both strands using the Big Dye Terminator v1.1 Cycle Sequencing kit (Applied Biosystems). Sanger sequencing was performed on ABI 3730 Genetic Analyzer (Applied Biosystems).

### Whole-transcriptome paired-end RNA sequencing

Total RNA was extracted from tumor specimens with RNeasy Mini Kit (Qiagen, Milan, Italy), then cDNA libraries were synthesized from 250 ng total RNA with TruSeq RNA Sample Prep Kit v2 (Illumina, San Diego, CA) according to the manufacturer’s recommendations. Sequencing by synthesis was performed on HiScanSQ sequencer (Illumina) at 75 bp in paired-end mode. Whole-transcriptome sequencing yielded an average of 61 million mapped reads/patient, thus reaching an average coverage of 44X. Two *SDHA*^mut^ tumor specimens were previously analyzed by whole transcriptome sequencing at the Genome Sciences Centre (Vancouver, Canada) [9].

### Bioinformatic analysis

After demultiplexing and FASTQ generation (both steps performed with Casava1.8, an application software specifically developed by Illumina), the paired-end reads quality were analyzed with the function fastx_quality_stats (part of FASTX Toolkit available at http://hannonlab.cshl.edu/fastx_toolkit/index.html). Based on these results we decided to trim each read of each sample at 74 bp in order to maximize sequence quality. The paired-end reads were mapped with the pipeline TopHat/Bowtie [61] on human reference genome HG19, collected from UCSC Genome Browser (http://www.genome.ucsc.edu/). After the alignment procedure the BAM file obtained was manipulated with Samtools [62] in order to remove the optical/PCR duplicate, to sort and to index it.

The analysis of gene expression was performed in two steps: 1) the function htseq-count (Python package HTseq) [63] was adopted to count the number of reads mapped on known genes, included in the Ensembl release 72 annotation features (http://www.ensembl.org); 2) the differential expressed genes were evaluated using the R-Bioconductor package edger [64]. DeFuse, ChimeraScan and FusionMap packages were used to detect chimeric transcripts from RNA-seq data.

### Gene expression analysis

RNA was extracted using RNeasy Mini Kit (Qiagen), quality-controlled and labeled as directed by the Affymetrix expression technical manual before hybridization to U133Plus 2.0 arrays. Gene expression data were quantified by the RMA algorithm, filtered and analyzed with supervised techniques by Limma modified t-test for the detection of differentially expressed genes. Differential expressed genes hierarchical clustering and Principal Component Analysis (PCA) were performed with Multiple Array Viewer (MEV available at http://www.tm4.org/mev.html). The same software was used to represent the data in the Figure 3 and Figure 4. Gene expression data of *KIT/PDGFRA*-mutated and *SDHA*-mutated samples were previously reported [30].

### Copy number analysis

Genomic DNA was labelled and hybridized to SNP array Genome Wide SNP 6.0 (Affymetrix) following manufacturer’s instructions. Quality control was performed by Contrast QC and MAPD calculation. Copy number analysis was performed by Genotyping Console and visualized with Chromosome Analysis Suite (*ChAS*) Software (Affymetrix). Hidden Markov Model algorithm was used to detect amplified and deleted segments with stringent parameters. To control for hyperfragmentation adjacent segments separated by < 50 probes were combined into one single segment, and only segments > 100 probes were considered.

### Quantitative PCR (qPCR)

Total RNA was reverse transcribed using Transcriptor First Strand cDNA synthesis kit (Roche Applied Science, Monza, Italy) with oligo-dT primers, according to the manufacturer’s guidelines. Gene-specific primers were designed with Primer Express 3.0 Software (Applied Biosystems) and qPCR was performed using FastStart Sybr Green (Roche) on the LightCycler 480 apparatus (Roche). DDCt method was used to quantify gene product levels relative to the GAPDH and ATP5B housekeeping genes. Significance was estimated by the Student’s *t* test: * p-value < 0.05; ** p-value < 0.01, *** p-value < 0.01.

### Western blot

Protein expression of *NTRK2* was evaluated on 2 *KIT*^WT^/*PDGFRA*^WT^/*SDH*^WT^/*RAS-P*^WT^ GIST and 8 *KIT* or *PDGFRA* or *SDH* mutant GISTs, of which fresh-frozen tissues were available. Tissue were disrupted in RIPA buffer (Sigma-Aldrich) supplemented with proteases inhibitors and lysed for 1 h with gentle agitation at 4°C. Lysates were centrifuged at 13,000 × *g* for 15 min at 4°C and supernatants were stored at −80°C. Protein concentrations were determined with the BCA protein assay (Pierce, Rockford, IL). Twenty micrograms of protein were resolved on a 8% SDS-PAGE gel and transferred onto polyvinylidene difluoride (PVDF) membranes. Nonspecific binding sites were blocked by incubation in blocking buffer (PBS containing 0.1% Tween-20 with 5% w/v milk) for 1 h at room temperature. Membranes were incubated overnight at 4°C, with the following primary antibodies: rabbit polyclonal *TRKB* antibody (ab18987 Abcam 1:500), and rabbit polyclonal β-*Tubulin* antibody (sc-9104 Santa Cruz Biotechnology, Santa Cruz, CA, 1:500). Then, membranes were washed and incubated with peroxidase conjugate secondary antibodies for 1 h at room temperature. Antigens were revealed using Enhanced Chemiluminescence Reaction (ECL Select, Amersham Pharmacia Biotech, Les Ulis, France).

## Nomenclature

*KIT*^WT^ No mutations of *KIT*

*PDGFRA*^WT^ No mutations of *PDGFRA*

*SDH*^WT^ No abnormalities of *SDHA/B/C/D* protein expression and/or gene mutation

*SDHA*^IHC –^ No expression of *SDHA* protein

*SDHA*^IHC +^ Normal expression of *SDHA* protein

*SDHB*^IHC –^ No expression of *SDHB* protein

*SDHB*^IHC +^ Normal expression of *SDHB* protein

*SDHA*^mut^ Mutation of *SDHA* protein (homozygous or compound heterozygote)

*SDHB*^mut^ Mutation of *SDHB* protein (homozygous or compound heterozygote)

*SDHC*^mut^ Mutation of *SDHC* protein (homozygous or compound heterozygote)

*SDHD*^mut –^ Mutation of *SDHD* protein (homozygous or compound heterozygote)

## Electronic supplementary material

Additional file 1: Table S1: *NTRK2* protein overexpression in *KIT* ^WT^/*PDGFRA* ^WT^/*SDH* ^WT^/*RAS-P* ^WT^ GIST. Western blot immunostaining of *NTRK2* was perfomed on proteins extracted from two *quadruple* ^WT^ GIST and from eight *PDGFRA* or *KIT* or *SDH* mutated GIST. HL-60 cell line protein extract was used as positive control. (XLSX 18 KB)

Additional file 2: Figure S1: *NTRK2* protein overexpression in *KIT* ^WT^/*PDGFRA* ^WT^/*SDH* ^WT^/*RAS-P* ^WT^ GIST. Western blot immunostaining of *NTRK2* was perfomed on proteins extracted from two *quadruple* ^WT^ GIST and from eight *PDGFRA* or *KIT* or *SDH* mutated GIST. HL-60 cell line protein extract was used as positive control. (DOCX 159 KB)

## Abbreviations

*CGRP*: Calcitonin gene-related peptide
CSS: Carney-Stratakis Syndrome
CT: Carney Triad
*DOG1*: Discovered on gastrointestinal stromal tumours 1
*ETS*: Erythroblast transformation-specific
GIST: Gastrointestinal stromal tumors
ICCs: Cells of Cajal
*IGF1R*: Insulin growth factor 1 receptor
IHC: Immunohistochemistry
*NF1*: Neurofibromatosis type 1
*PDGFRA*: Platelet-derived growth factor receptor alpha
*RAS*-P: RAS-pathway
*SDH*: Succinate dehydrogenase
SNV: Single nucleotide variant
WT: Wild-type.
