# Supplementary material for: Integrated genomic study of quadruple-WT GIST (KIT/PDGFRA/SDH/RAS pathway wild-type GIST)
Source: BMC Cancer. 2014 Sep 20;14:685. doi: 10.1186/1471-2407-14-685 (PMC4181714; doi:10.1186/1471-2407-14-685)
Supplement: Supplementary file 2 — Additional file 2: Figure S1: NTRK2 protein overexpression in KIT WT/PDGFRA WT/SDH WT/RAS-P WT GIST. Western blot immunostaining of NTRK2 was perfomed on proteins extracted from two quadruple WT GIST and from eight PDGFRA or KIT or SDH mutated GIST. HL-60 cell line protein extract was used as positive control. (DOCX 159 KB) [file 12885_2014_4877_MOESM2_ESM.docx]

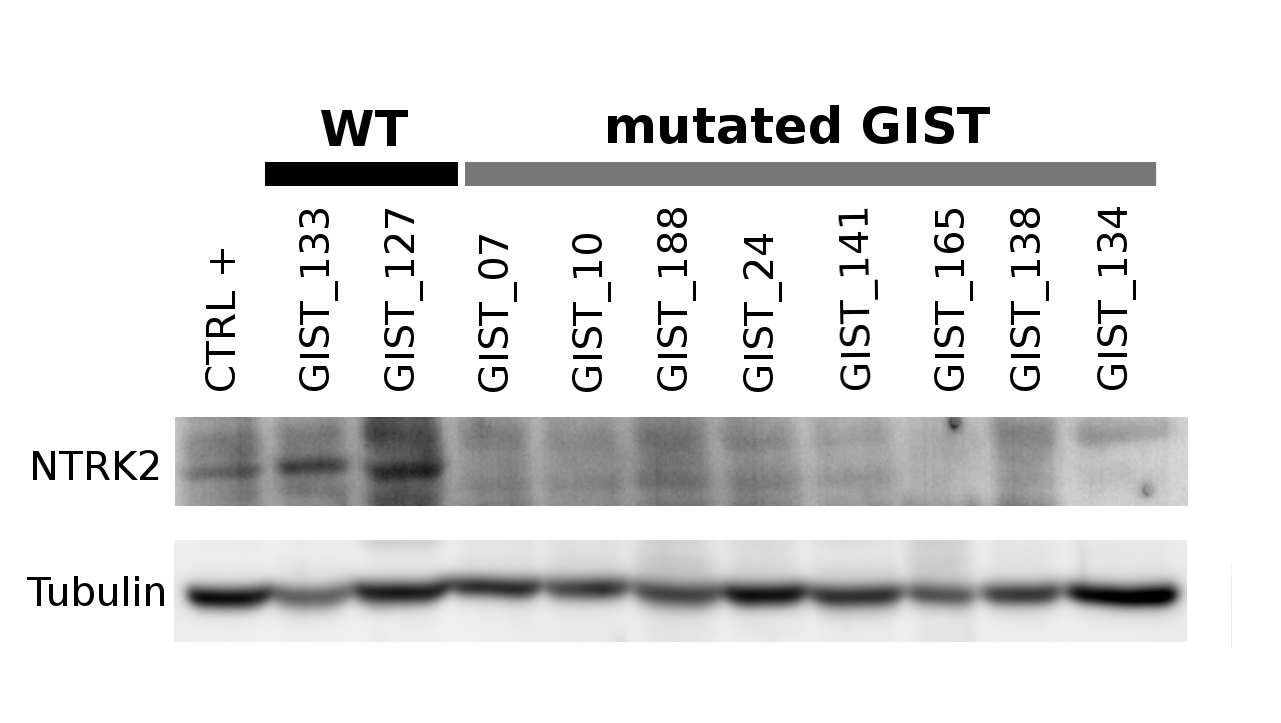


**Supplementary Figure 1.** *NTRK2* protein overexpression in *KIT*^WT^/*PDGFRA*^WT^/*SDH*^WT^/*RAS-P*^WT^ GIST. Western blot immunostaining of *NTRK2* was perfomed on proteins extracted from two *quadruple*^WT^ GIST and from eight *PDGFRA* or *KIT* or *SDH* mutated GIST. HL-60 cell line protein extract was used as positive control.
